# PREDICTIONS FOR DEMENTIA PREVALENCE IN CHINA BY 2050: A MARKOV MODELING STUDY

**DOI:** 10.1093/geroni/igad104.0648

**Published:** 2023-12-21

## Abstract

Dementia brings a heavy burden on older adults, their family and the society at large. Accurate prediction of dementia prevlance is important for preventive strategy for fast- ageing China. The purpose of this study is to predict dementia prevalence in China by 2050, taking into account variations in dementia incidence rates and mortality rates in future. Based on two nationally representative Chinese ageing cohorts (i.e. CLHLS, CHARLS), a ten-state Markov model (IMPACT-CAM), including prevalence, transition probability and mortality rate of dementia and its associated cardiovaiscualr diseases and disability, was constructed to predict dementia prevalence in people aged above 60 years by 2050, with different assumptions on the furture trends of dementia incidence and mortality rates. IMPACT-CAM projected there were approximately 57.9 million (95% uncententiy interval 56.3-59.6 m) people with dementia by 2050, assuming constant incidence rate of dementia and declining mortality rates over coming years. In comparison with this assumption, if the incidence rate of dementia decreasing by 1.0% annually coupled with declining mortality rates, the projected number of dementia cases would be 9.8 million less; if dementia incidence and mortality rates both remained constant since 2022, the projection would be 18.5 million less. The estimated dementia prevlance for people aged 60 years in 2050 were 12.0%, 9.9% and 9.5%, respectively. Alongside the irreversible population ageing trend, the number of people with dementia in China is likely to increase rapidly in the near furture. Nevertheless, effective preventing measures of dementia would restrain the surge substantially.

